# Dirac cone in ***α***-graphdiyne: a first-principles study

**DOI:** 10.1186/1556-276X-8-469

**Published:** 2013-11-09

**Authors:** Xiaoning Niu, Xingze Mao, Dezheng Yang, Zhiya Zhang, Mingsu Si, Desheng Xue

**Affiliations:** 1 Key Laboratory for Magnetism and Magnetic Materials of the Ministry of Education, Lanzhou University, 222 South Tianshui Road, Lanzhou 730000, China

**Keywords:** *α*-graphdiyne, Dirac cone, First-principles calculation

## Abstract

We investigate the Dirac cone in *α*-graphdiyne, which is a predicted flat one-atom-thick allotrope of carbon using first-principles calculations. *α*-graphdiyne is derived from graphene where two acetylenic linkages (-C ≡C-) are inserted into the single bonds (-C-C-). Thus, *α*-graphdiyne possesses a larger lattice constant which subsequently affects its electronic properties. Band structures show that *α*-graphdiyne exhibits similar Dirac points and cone to graphene. Further, the tight-binding method is used to exploit the linear dispersion in the vicinity of Dirac points. Thanks to the larger lattice constant, *α*-graphdiyne yields a lower Fermi velocity, which might make itself an ideal material to serve the anomalous integer quantum Hall effect.

## Background

Band theory was first used to study the band structure of graphene over half a century ago [1], and it demonstrated that graphene is a semimetal with unusual linearly dispersing electronic excitations called Dirac electron. Such linear dispersion is similar to photons which cannot be described by the Schrödinger equation. In the vicinity of the Dirac point where two bands touch each other at the Fermi energy level, the Hamiltonian obeys the two-dimensional (2D) Dirac equation [2] as $H=v_{F}\vec{\sigma }\cdot \hat{p}$ with *v*_F_ being the Fermi velocity, $\vec{\sigma }$ the Pauli matrices, and $\hat{p}$ the momentum operator. In graphene, the Fermi velocity *v*_F_ is 300 times smaller than the speed of light. Hence, many unusual phenomena of quantum electrodynamics can be easily detected because of the much lower speed of carriers [3]. Within the framework of tight-binding approximation, the Fermi velocity *v*_F_ is proved to be dependent on both the lattice constant and the hopping energy. In fact, the hopping energy is also associated with the lattice constant. Thus, the Fermi velocity of Dirac cone materials might be tunable through changing the corresponding lattice constant.

Recently, it was found that Dirac cones not only occur in the 2D carbon allotropes such as graphene, graphyne, and graphdiyne [4], but also can be detected at interfaces of topological insulators [5-11]. It is notable that, in 6,6,12-graphyne [4], the conduction electrons turn out to be superior to that in graphene in one preferred direction over the other, which is due to the rectangular lattice. This is a major step in searching for new Dirac cone materials. Therefore, it is proper to pursue the Dirac cone material with tunable Fermi velocity, which will be the focus of future researches.

In this letter, we predict a novel flat one-atom-thick allotrope of carbon by inserting two acetylenic linkages into the single bonds in graphene. According to the naming method used in [4], we assign it as *α*-graphdiyne. Up to now, no study has been made on *α*-graphdiyne both experimentally and theoretically. Thus, theoretical investigation on *α*-graphdiyne is a must before synthesizing it in experiments. Since *α*-graphdiyne has a larger lattice constant, it should have potential applications both in quantum tunneling [12] and in anomalous integer quantum Hall effect [13]. In this work, band structures are calculated and a similar Dirac cone to that of graphene is observed. In particular, we introduce a tight-binding model to mimic the hopping energy between the hexagonal vertices, which realizes the linear dispersion of bands near the Dirac points, allowing the Dirac cone to be studied explicitly.

## Methods

To simulate the electronic properties, we employ density functional theory with the generalized gradient approximation (GGA) of Perdew-Burke-Ernzerhof (PBE) [14] for the exchange-correlation (XC) potential within the projector augmented wave method, as implemented in VASP [15]. The cutoff energy for plane waves is set to be 500 eV. The vacuum space is at least 15 Å, which is large enough to avoid the interaction between periodical images; 15 ×15×1 and 25 ×25×1 are used for the **k**-grid of geometry optimization and self-consistent calculation, respectively. During the geometry optimization, all the atoms in the unit cell were allowed to relax and the convergence of force is set to 0.001 eV/Å.

## Results and discussion

**Figure 1:**
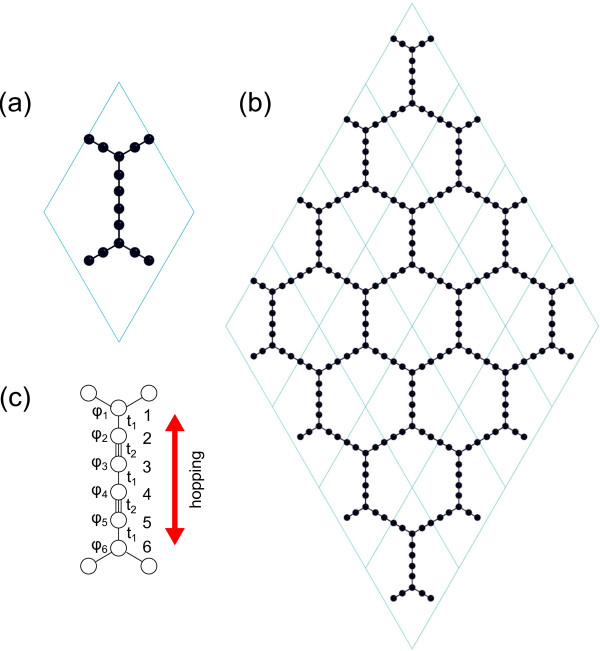
**Crystal structure of *α*-graphdiyne.(a)** A unit cell and **(b)** a 4×4 supercell. **(c)** A simplified model to mimic the hopping matrix elements along two carbon triple bonds in *α*-graphdiyne. Carbon atoms 1 and 6 are at vertices of a hexagon in *α*-graphdiyne. The black balls and blue line represent carbon atoms and the crystalline cell, respectively.

Based on first-principles calculation, the lattice structure of *α*-graphdiyne is predicted for the first time, as shown in Figure 1. It clearly shows that *α*-graphdiyne has a hexagonal lattice the same as graphene. The optimized lattice constant is 11.42 Å. This is very insightful. On one hand, it has the largest lattice constant compared with currently known carbon allotropes [16] and thus has a much smaller density than graphene and other related carbon allotropes. This makes *α*-graphdiyne a potential candidate for hydrogen storage [17]. At the same time, the absorbed hydrogen may induce an intrinsic magnetism in the defected system [18,19]. On the other hand, this lattice constant has a very little mismatch with Si(111) surface, that is, one unit cell of *α*-graphdiyne matches the Si(111)-3 ×3 unit cell well. This suggests that the high possibility is to grow *α*-graphdiyne epitaxially on Si(111) substrate. After the epitaxial structure is cooled down, one can remove the substrate by chemical etching. In this way, the isolation of monolayer *α*-graphdiyne might be obtained in experiments.

**Figure 2:**
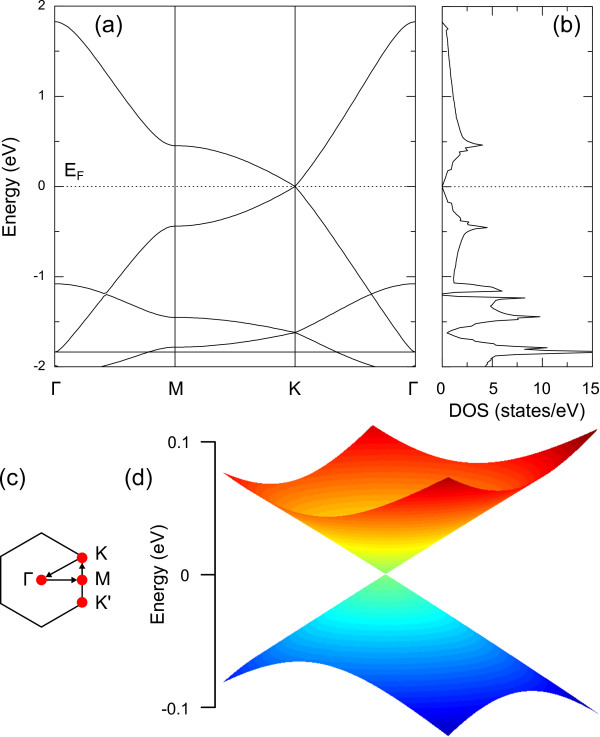
**Electronic properties of *α*-graphdiyne.(a)** Band structure and **(b)** DOS. **(c)** First Brillouin zone with the letters designating high-symmetry points. **(d)** 2D Dirac cone representing the valence and conduction bands in the vicinity of the *K* and *K*^′^ points. *E*_F_ is the Fermi energy.

The band structure and density of states (DOS) of *α*-graphdiyne are shown in Figure 2a,b, respectively. The most important observations from Figure 2a are the linear dispersion near the *K* point and the zero DOS at the Fermi energy level. However, the corresponding slope of the Dirac cone is obviously smaller than that of graphene and *α*-graphyne. This has a big effect on the Fermi velocity, as discussed below. The bonding and antibonding orbitals at the Fermi energy level touch each other and develop two slight flat bands as *K* approaches *M*, which correspond to the two peaks near the Fermi level in the DOS plot. Similar to the case of graphene and *α*-graphyne, the Dirac points are located at the *K* and *K*^′^, which means that there are even (six) Dirac points in the Brillouin zone, which is in a striking difference from the odd Dirac points observed in topological insulator Bi_2_Te_3_[20].

Due to the breaking symmetry associated with spin-orbit interaction (SOI) in 2D layered materials, a small band gap will be induced at the Dirac points, which can in principle be used to study the quantum spin Hall effect. The energy bands with SOI (not shown for brevity) open a band gap of 22 ×10^-3^ meV in *α*-graphdiyne, and the magnitude is close to the value of graphene [21].

To understand the nature of the Dirac cone in *α*-graphdiyne, we employ the tight-binding method proposed in [22], where an effective hopping parameter is introduced. It is notable that there are six carbon atoms along the effective hopping direction in *α*-graphdiyne, as shown in Figure 1, while only four in *α*-graphyne. This makes it more complex to exploit *α*-graphdiyne than *α*-graphyne. To simplify the model, two triple bonds with the hopping parameters *t*_1_ and *t*_2_ for the single and triple carbon bonds are taken. The simplified Hamiltonian equations at the carbon triple bond, i.e., sites 2, 3, 4, and 5, are

(1) $$\begin{array}{c} E\varphi_{2}=V\varphi_{2}+t_{1}\varphi_{1}+t_{2}\varphi_{3},\\ E\varphi_{3}=V\varphi_{3}+t_{2}\varphi_{2}+t_{1}\varphi_{4},\\ E\varphi_{4}=V\varphi_{4}+t_{1}\varphi_{3}+t_{2}\varphi_{5},\\ E\varphi_{5}=V\varphi_{5}+t_{2}\varphi_{4}+t_{1}\varphi_{6}, \end{array}$$

where *E* and *V* are the electron and on-site energies, respectively. Based on Equation 1, we can easily obtain

(2) $$\begin{array}{c} \varphi_{2}=\frac{t_{1}(E-V)\varphi_{1}+t_{1}t_{2}\varphi_{4}}{{\left(E-V\right)}^{2}-t_{2}^{2}},\\ \varphi_{3}=\frac{t_{1}t_{2}\varphi_{1}+t_{1}(E-V)\varphi_{4}}{{\left(E-V\right)}^{2}-t_{2}^{2}},\\ \varphi_{4}=\frac{t_{1}(E-V)\varphi_{3}+t_{1}t_{2}\varphi_{6}}{{\left(E-V\right)}^{2}-t_{2}^{2}},\\ \varphi_{5}=\frac{t_{1}t_{2}\varphi_{3}+t_{1}(E-V)\varphi_{6}}{{\left(E-V\right)}^{2}-t_{2}^{2}}. \end{array}$$

When we focus on the electrons near the Dirac cone where *E* ≈ *V*, the wave functions are approximately *φ*_2_ ≅ -(*t*_1_/*t*_2_)*φ*_4_, *φ*_3_ ≅ -(*t*_1_/*t*_2_)*φ*_1_, *φ*_4_ ≅ -(*t*_1_/*t*_2_)*φ*_6_, and *φ*_5_ ≅ -(*t*_1_/*t*_2_)*φ*_3_. Thus, the hopping term from site 2 to 1 is $t_{1}\varphi_{2}≅-(t_{1}^{2}/t_{2})\varphi_{4}$, from site 3 to 4 is $t_{1}\varphi_{3}≅-(t_{1}^{2}/t_{2})\varphi_{1}$, from site 4 to 3 is $t_{1}\varphi_{4}≅-(t_{1}^{2}/t_{2})\varphi_{6}$, and from site 5 to 6 is $t_{1}\varphi_{5}≅-(t_{1}^{2}/t_{2})\varphi_{3}$. With the above four hopping terms, we thus have

(3) $$\begin{array}{c} t_{1}\varphi_{2}≅(t_{1}^{3}/t_{2}^{2})\varphi_{6}\equiv {\tilde{t}}_{1}\varphi_{6},\\ t_{1}\varphi_{5}≅(t_{1}^{3}/t_{2}^{2})\varphi_{1}\equiv {\tilde{t}}_{1}\varphi_{1}, \end{array}$$

which means that the effective direct hopping parameter between sites 1 and 6 is

(4) $${\tilde{t}}_{1}\equiv (t_{1}^{3}/t_{2}^{2}).$$

The obtained effective hopping parameter ${\tilde{t}}_{1}$ has the same sign as *t*_1_, which means that pseudospin in *α*-graphdiyne has the same direction as in graphene. Thus, many perspectives of graphene can be transferred to *α*-graphdiyne directly. The magnitude of ${\tilde{t}}_{1}$ depends on the hopping parameter *t*_2_. Remarkably, it equals *t*_1_/*t*_2_ times the effective hopping parameter in *α*-graphyne. Thus, the effective hopping parameter should be smaller in *α*-graphdiyne than in *α*-graphyne as *t*_1_/*t*_2_ < 1.

Once we obtain the effective hopping parameter ${\tilde{t}}_{1}$, the standard energy-momentum relation can be obtained directly as [1]

(5) $$\begin{array}{c} E(k_{x},k_{y})=\pm {\tilde{t}}_{1}\sqrt{1+4\text{cos}(\frac{\sqrt{3}}{2}k_{x}a)\text{cos}(\frac{1}{2}k_{y}a)+4{\text{cos}}^{2}(\frac{1}{2}k_{y}a)}, \end{array}$$

where *a* is the lattice constant. By fitting the occupied and unoccupied bands in the vicinity of the *K* point from the first-principles calculations, as illustrated in Figure 2a, the renormalized hopping parameter ${\tilde{t}}_{1}$ has a value of 0.45 eV. It is much smaller than the value of approximately 3 eV in graphene, which originates from the larger lattice constant in *α*-graphdiyne. Figure 2c shows the high-symmetry points in the first Brillouin zone. It explicitly shows that the energy bands are degenerate to zero at both *K* and *K*^′^ points. In Figure 2d, a 2D plot of the Dirac cone of *α*-graphdiyne is displayed. Due to the same hexagonal lattice as graphene and *α*-graphyne, the 2D Dirac cone of *α*-graphdiyne exhibits a similar appearance.

It is known that the Fermi velocity plays a vital role in the photoelectric field and crucially dominates the transport properties. Here, we will focus attention on the study of Fermi velocity of *α*-graphdiyne. The dispersion close to the *K* and *K*^′^ points can be expanded as

(6) $$E_{\pm }(\text{q})\approx \pm v_{F}|\text{q}|+O[\;|\text{q}{|}^{2}],$$

where **q** is the momentum measured relative to the Dirac points, ‘ ±’ the upper and lower Dirac cones, and *v*_F_ the Fermi velocity, given by $v_{F}=\sqrt{3}\text{ta}/2$. With the lattice constant *a* = 11.42 Å and the effective hopping parameter ${\tilde{t}}_{1}$ = 0.45 eV, the slope of the Dirac cone in *α*-graphdiyne equals ±4.5 eVÅ compared with ±28 eVÅ in *α*-graphyne and ±34 eVÅ in graphene [4]. The corresponding Fermi velocity is about 0.11 ×10^6^ m/s, which is much lower than the value in *α*-graphyne. From this perspective, *α*-graphdiyne, which has a lower Fermi velocity than other known carbon allotropes, will lead to possible applications in quantum electrodynamics, for example, to observe the anomalous integer quantum Hall effect at room temperature [13].

**Figure 3:**
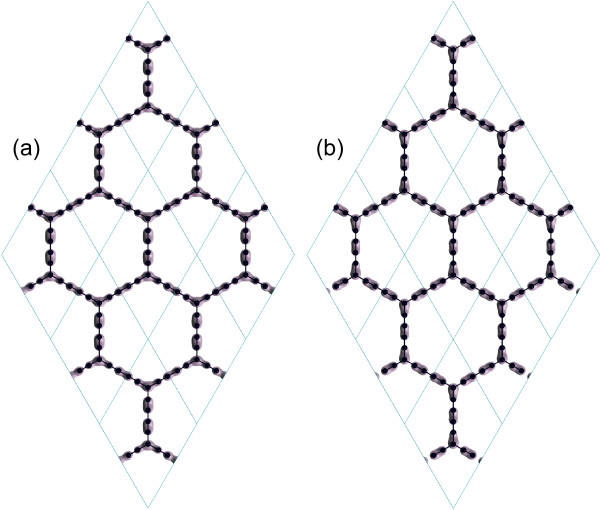
**Charge density distributions of two orbitals at the Dirac point.** The **(a)** bonding and **(b)** antibonding bonds. The isovalues are set to 0.03 Å ^-3^; 3 ×3 supercells are given for the sake of clarity.

More information including the helical texture of Dirac cone and Berry’s phase are indeed associated with the detailed wave functions. In this work, we instead calculate the two orbitals at the Dirac point as shown in Figure 3. The charge density explicitly exhibits a 180° rotational symmetry, which is consistent with the theoretical conclusion in the literature [2]. The two orbitals consist of two types of bonds in *α*-graphdiyne: One is the bonding bonds (Figure 3a) and the other the antibonding bonds (Figure 3b), which are located at the different carbons. As a recent study reported [23], the effective hopping term of the acetylenic linkages is much smaller than the direct hopping between the vertex atoms. This is because the covalent bonds are formed in these acetylenic linkages as illustrated in Figure 3, which subsequently weakens the hopping ability. Thus, the reduced hopping parameter is a natural consequence, which also agrees well with our above tight-binding theory. Future experiments can test this prediction directly.

## Conclusions

In conclusion, we have predicted a novel carbon allotrope called *α*-graphdiyne, which has a similar Dirac cone to that of graphene. The lower Fermi velocity stems from its largest lattice constant compared with other current carbon allotropes. The effective hopping parameter ${\tilde{t}}_{1}$ of 0.45 eV is obtained through fitting the energy bands in the vicinity of Dirac points. The obtained Fermi velocity has a lower value of 0.11 ×10^6^ m/s, which might have potential applications in quantum electrodynamics.

## Competing interests

The authors declare that they have no competing interests.

## Authors’ contributions

MSS designed the work and revised the paper. XNN calculated the first-principles results. XZM wrote the manuscript. DZY, ZYZ, and DSX have devoted valuable discussion. All authors read and approved the final manuscript.
